# Meningeal Carcinomatosis: A Metastasis from Gastroesophageal Junction Adenocarcinoma

**DOI:** 10.1155/2013/245654

**Published:** 2013-12-19

**Authors:** Tanya Dam, Aftab Mahmood, Kenneth Linville, Michael Bailey, Salim Surani

**Affiliations:** ^1^Bay Area Medical Center, 109 Catawba Trc, Morgantown, NC 28655, USA; ^2^Bay Area Medical Center, 1625 Rodd Field Road, Corpus Christi, TX 78412, USA; ^3^Bay Area Medical Center, 7101 South Padre Island Drive, Corpus Christi, TX 78412, USA; ^4^Texas A&M University, 1177 West Wheeler Avenue, Suite 1, Aransas Pass, TX 78336, USA

## Abstract

Gastroesophageal adenocarcinoma is a malignant type of cancer, which can metastasize to multiple organs. However, there have not been many case reports in the literature pertaining the relationship of gastroesophageal adenocarcinoma and carcinomatous meningitis. In this case, a 65-year-old African American male with a history of dysphagia was initially diagnosed with adenocarcinoma at gastroesophageal junction. The patient was treated with both chemotherapy and radiation, but chemotherapy was interrupted due to significant weight loss, anemia, and sudden onset of change in mental status. Patient was admitted to our facility for further evaluation of his neurological symptoms. The patient became more confused and delirious during hospital stay, and symptoms could not be explained by radiological studies and laboratory values. Therefore, a lumbar puncture was done to search for infectious and neoplastic causes that were not shown up on Computed Tomography scan (CT) and Magnetic Resonance Imaging scan (MRI) of the brain. The cerebrospinal fluid (CSF) cytology showed metastatic poorly differentiated adenocarcinoma. The patient's prognosis was poor because there is no specific treatment recommendation for primary gastroesophageal cancer at this stage. The patient passed away 4 weeks later under hospice care. The goal of our case report is to raise awareness of the rare metastatic possibility in advanced stage of gastroesophageal adenocarcinoma. In doing so, physicians can help educate and prepare family for unfavorable outcomes.

## 1. Introduction

Carcinomatous meningitis consists of metastatic tumor cells involving the leptomeninges and the circulating cerebrospinal fluid; albeit, it is an uncommon complication of solid tumors. It occurs in 5–10% of patients with breast cancer, lung cancer, or melanoma [1]. It is extremely rare in patients with gastrointestinal malignancy. In fact, brain metastases from esophageal carcinoma were found in only 36 patients between 1986 and 2000 in Japan [2]. A review of the literature found 3 case reports before 2002 and 8 cases in 2003 [3]. A retrospective study from 1944 to 2002 revealed 21 patients with gastrointestinal primary tumors and neoplastic meningitis. The frequency of leptomeningeal involvement in gastric adenocarcinoma was 8 out of 5618 patients (0.14%) and 7 out of 4361 (0.16%) in esophageal adenocarcinoma [1]. We performed a recent search of Pub Med and found 2 more case reports on leptomeningeal carcinomatosis secondary to gastroesophageal adenocarcinoma in 2011 [4]. We hereby present an additional case of leptomeningeal carcinomatosis from gastroesophageal junction (GE) adenocarcinoma.

## 2. Case Report

A 65-year-old African American male presented with a 4-month history of dysphagia and 30-pound unintentional weight loss in an outpatient clinic. The patient underwent an endoscopy, which showed a friable, nodular, and circumferential, near obstructing mass extending from the distal esophagus into the gastric fundus. The biopsy was interpreted as well-differentiated adenocarcinoma. Staging CT scans of chest, abdomen, and pelvis confirmed a large mass near the GE junction measuring 6 cm in size accompanied by enlarged pancreaticoduodenal lymph nodes in the vicinity of the gastric cardia. There were no distant metastases noted. The patient underwent a Positron emission tomography scan (PET) which showed a hypermetabolic gastric mass. There was focal uptake in the dome of the liver and probable avascular necrosis of the hips, but follow-up MRI of the liver showed no liver metastases.

After declining surgery and percutaneous endoscopic gastrostomy tube (PEG) placement, the patient received two cycles of chemotherapy with Cisplatin and 5-Fluorouracil and then began concurrent chemotherapy and radiation therapy. The therapeutic regimen was complicated by severe anemia with hemoglobin of 6.5 g/dL and hematocrit of 24%, which required transfusion. The patient also developed increased dysphagia, dehydration, and malnutrition resulting in another 30-pound weight loss over 4 weeks. Therefore, the patient left in the middle of chemotherapy treatment. Two months later, the patient relocated to a different city and was seen by a new oncologist.

In the new cancer center, a following up PET scan was obtained which showed neoplasm in the gastric cardia and possible liver metastasis but no brain metastasis. A repeat CT scan of the abdomen with and without contrast confirmed again no focal liver lesion. The patient initially declined chemotherapy but agreed to complete radiation therapy. The 6 cm gastric tumor was radiated again palliatively at this point with hope to improve his oral nutritional intake. However, the patient continued to have a 10-pound weight loss and normocytic anemia with hemoglobin 7.9 g/dL, hematocrit 24.8%, and MCV 88 fL.

The patient then developed sudden change in mental status two weeks after completion radiation therapy. The patient was admitted to our facility and found to have acute renal failure with creatinine 2.4 mg/dL and urinary tract infection with WBC 15.6/mm^3^. Urine culture reported pseudomonas, so the patient had double antibiotic coverage with ciprofloxacin and ceftazidime based on culture sensitivity. Bilateral renal ultrasounds and CT abdomen and pelvis without contrast confirmed mild bilateral hydronephrosis. The radiology findings revealed retroperitoneal lymphadenopathy, which resulted in proximal bilateral ureteric obstruction. There were calcified lymph nodes in the porta hepatis and lesser sac region. In addition, CT and MRI scans of the head were necessitated by neurologic symptoms and suggested communicating hydrocephalus.

**Figure 1:**
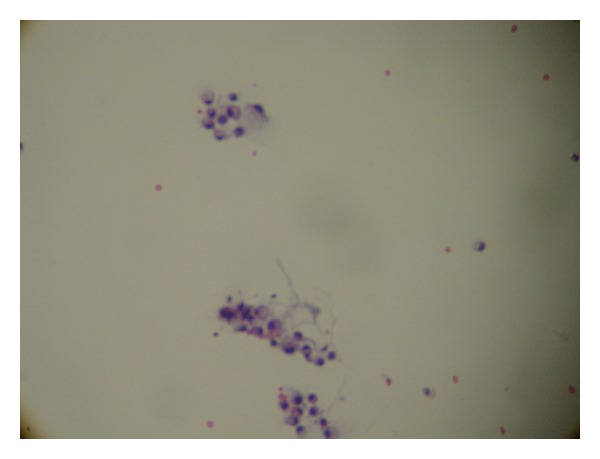
Cytology of cerebral spinal fluid showing clusters of adenocarcinoma cells.

**Figure 2:**
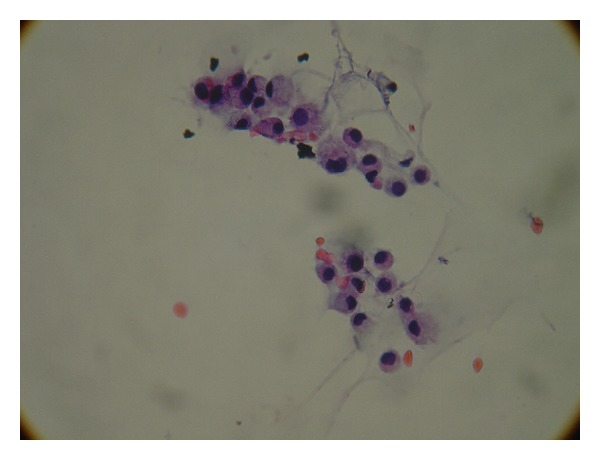
High power view of adenocarcinoma cells in cerebral spinal fluid.

**Figure 3:**
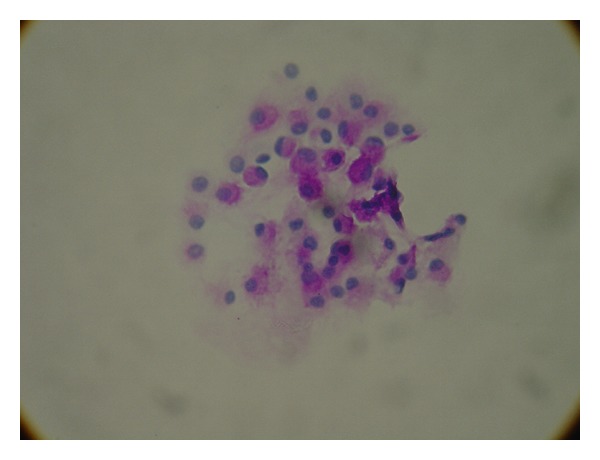
A Periodic acid-Schiff (PAS) stain showing adenocarcinoma cells with pink staining of mucin in the cytoplasm.

A neurologist was consulted who performed a lumbar puncture revealing an opening pressure of 27 mmHg, closing pressure of 9 mmHg, WBC 11/mm^3^, and RBC 4/mm^3^. Glucose and protein levels were within normal limits. Gram stain and spinal fluid culture revealed no growth. The patient had no cognitive improvement after therapeutic spinal fluid drainage. However, the cytology returned with remarkable metastatic poorly differentiated adenocarcinoma as shown in Figures 1, 2, and 3.

At this stage, there was no effective treatment for brain metastasis from gastric adenocarcinoma. Cranial and whole brain radiation were options, which might alleviate pain and decrease development of new neurologic symptoms. Family members did not want any more radiation treatment because patient had a difficult time with gastric mass radiation two weeks before hospitalization. The prognosis being poor and low 6-month survival rate, the family elected to place the patient on hospice care. He passed away 4 weeks later.

## 3. Discussion

Carcinomatous meningitis secondary to GE junction adenocarcinoma is rare; therefore, it is difficult to diagnose. Head CT and MRI tests were commonly ordered, but the sensitivity was reported as 76% in Straathof et al. study [5]. Therefore, the test of choice was lumbar puncture, which had high sensitivity up to 91% on repeated procedure according to Wasserstrom et al. [6]. To minimize false negative results from lumbar puncture, Yamada et al. recommended collection of minimum 10 mL of CSF fluid, immediate ethanol-based fixation for cytology, CSF from a site of known leptomeningeal disease and repeated lumbar puncture if initial cytology is negative but clinical suspicion is high [7]. In our case, we were able to make the diagnosis from the first set of CSF fluid when all imaging modalities failed to explain clinical symptoms.

Gastroesophageal junction (GE) adenocarcinoma is known as one of the most difficult malignancies to treat. The development of carcinomatous meningitis from the GE junction primary site bespeaks the poor prognosis with survival averaging 4–16 weeks after diagnosis of metastasis [1]. There is no effective treatment, but current available options are corticosteroids, radiation therapy, and chemotherapy. Corticosteroids do not reverse neurologic deficits but can improve headache and pain. Radiation therapy includes cranial irradiation and whole brain radiation to target symptomatic sites for palliative purpose. In our case, malignant cells in the subarachnoid space could obstruct normal CSF reabsorption pathways via arachnoid granulations, which resulted in communicating hydrocephalus. Radiation therapy might restore CSF flow if a focal area of malignancy cells would be located. Before radiation, a cisternogram could be obtained with tagged indium 111 or DTPA tracers to determine if there was abnormal CSF flow within the brain and spinal canal. The CSF with tracers would be recorded with a gamma camera at 6 hours, 24 hours, 48 hours, and 72 hours. However, the test is labor intensive and requires cooperation from the patient. The test can suggest area of CSF flow disturbance but cannot quantify malignant cell burden inside CSF. In addition, even though whole brain radiation possibly improves CSF flow dynamics, it cannot reverse prior focal neurologic deficits.

The most aggressive treatment is intrathecal lumbar or intraventricular injection of chemotherapeutic drugs such as methotrexate and cytosine arabinoside or liposomal cytarabine. In a 2001 case report from Cezch Republic, a 39-year-old patient having meningeal carcinomatosis with gastric primary received spinal injection of cytosine arabinoside (Cytosar), methotrexate, and hydrocortisone. The patient died from multiple complications including disseminated intravascular coagulopathy and cerebral edema after 44 days of treatment [8]. Other four cases from France in 2001 were administered intrathecal methotrexate that achieved two to three month survival and improvement in neurologic symptoms [9]. In 2009, a retrospective study from United States by Oh et al. reported CSF cytology conversion from positive cytology to negative cytology after intrathecal chemotherapy that prolonged survival rate [10]. Another recently studied intrathecal chemotherapy agent was topotecan. In fact, a multicenter phase II trial of intrathecal topotecan was conducted at the University of Texas M.D Anderson Cancer Center in 2008. Only 13 out of 40 patients (21%) had CSF clearance of malignant cells after 6 weeks. The median survival rate was 15 weeks, so it provided no added benefit over other intrathecal drugs [11]. Intrathecal chemotherapy was only effective for small tumor cell deposits or floating malignant cells in CSF because chemodrugs would not be able to diffuse in tumor thicker than 1 mm [12]. The preferable routes of admission included subcutaneous reservoir and ventricular catheter (Ommaya device) rather than lumbar puncture for more uniform drug distribution [13]. Even though multiple recent studies demonstrated promising results, due to unclear survival benefit and limited data, intrathecal chemotherapy is still an area of research.

## 4. Conclusion

In conclusion, the incidence of brain metastasis from GE junction adenocarcinoma may increase in the future due to increasing survival time for patients treated with chemoradiation [3]. Physicians should be aware of this potential complication and followup with appropriate tests. In fact, no single test is reliable to detect it with complete certainty. When there is a high index of suspicion, both MRI and CSF cytology should be ordered to increase diagnostic efficacy [5]. Once the diagnosis is made, medical staff should work closely with patient and family to prepare for unfavorable outcomes. Managing poor-risk patients with encephalopathy, neurologic deficits, low performance status, and extensive systemic cancer, treatment should focus on palliative care and avoid intrathecal chemotherapy.
